# Supplementary material for: Robust subspace methods for outlier detection in genomic data circumvents the curse of dimensionality
Source: R Soc Open Sci. 2020 Feb 5;7(2):190714. doi: 10.1098/rsos.190714 (PMC7062061; doi:10.1098/rsos.190714)
Supplement: Supplmentary Material [file rsos190714supp1.pdf]

## 0.1 GOP Closed Form Solution for Iterative Steps

In this section we will derive the closed form expressions for the three update steps of the ADMM algorithm used to minimize the GOP objective function, Algorithm 2 in section 2.4 of the manuscript.
We rewrite the GOP objective function as follows:

$$\begin{aligned}&\min_{L,C,Q} ||L||_* + \lambda||C||_{1,2} + \alpha\text{tr}(Q\Phi Q^T)\\&\text{subject to} : M = L + C, \quad L = Q\end{aligned} \tag{1}$$

Now we can define the augmented Lagrangian function of 0.1:

$$\begin{aligned}&\mathcal{L}(L, C, Q, Z_1, Z_2) = ||L||_* + \lambda||C||_{1,2} + \alpha\text{tr}(Q\Phi Q^T)\\&+ \langle Z_1, M - L - C\rangle + \frac{r_1}{2}||M - L - C||_F^2\\&+ \langle Z_2, Q - L\rangle + \frac{r_2}{2}||Q - L||_F^2.\end{aligned}$$

Now we can divide the solution of this problem by iterating through three subproblems sequentially as shown in Algorithm 2 in main text. We need to solve the following three subproblems in this order:

1. $L^{k+1} = \underset{L}{\text{argmin}}\mathcal{L}(L, C^k, Q^k, Z_1^k, Z_2^k)$ .
2. $C^{k+1} = \underset{C}{\text{argmin}}\mathcal{L}(L^{k+1}, C, Q^k, Z_1^k, Z_2^k)$
3. $Q^{k+1} = \underset{Q}{\text{argmin}}\mathcal{L}(L^{k+1}, C^{k+1}, Q, Z_1^k, Z_2^k)$

the three subplroblems have closed form solutions. However before showing the updates of the primal variables $L, C, Q$ we need to define the proximity operator [1]. The proximity operator is defined by:

$$\text{prox}_h(X) = \underset{Y}{\text{argmin}}\, h(Y) + \frac{1}{2}||Y - X||_F^2 \tag{2}$$

where $h : \mathbb{R}^{p \,\text{x}\, n} \rightarrow \mathbb{R}$ is a convex function that takes as input a matrix with dimensions $p$ x $n$ and outputs a real valued number. Now we show the closed form solutions of the updates.

**Updating** $L$ (finding $L^{k+1}$):
$L^{k+1} = \underset{L}{\text{argmin}}\mathcal{L}(L, C^k, Q^k, Z_1^k, Z_2^k)$. terms that are not related to $L$ are constants and thus are discarded. This gives us:

$$L^{k+1} = \underset{L}{\operatorname{argmin}} ||L||_* + \langle Z_1^k, M - L - C^k \rangle + \frac{p_1}{2}||M - L - C^k||_F^2 + \langle Z_2^k, Q^k - L \rangle + \frac{p_2}{2}||Q^k - L||_F^2$$
$$= \underset{L}{\operatorname{argmin}} ||L||_* + \frac{p_1}{2}\Big|\Big|L - (M - C^k + \frac{Z_1^k}{p_1})\Big|\Big|_F^2 + \frac{p_2}{2}\Big|\Big|L - (Q^k + \frac{Z_2^k}{p_2})\Big|\Big|_F^2$$
$$= \underset{L}{\operatorname{argmin}} \frac{||L||_*}{p_1 + p_2} + \frac{1}{2}\Big|\Big|L - \frac{p_1 R_1^k + p_2 R_2^k}{p_1 + p_2}\Big|\Big|$$
$$= \operatorname{prox}_{\frac{||L||_*}{p_1+p_2}}(\frac{p_1 R_1^k + p_2 R_2^k}{p_1 + p_2}).$$

where $R_1^k = M - C^k + \frac{Z_1^k}{p_1}$ and $R_2^k = Q^k + \frac{Z_2^k}{p_2}$. The proximity operator of the nuclear norm function is the singular value soft-thresholding operator [2], which is defined as $\mathcal{D}_\epsilon(X) = U\xi_\epsilon(\Sigma)V^T$, where $X = U\Sigma V^T$ is the singular value decomposition (SVD) of X and $\xi_\epsilon(\Sigma)$ is the soft-thresholding operator on the diagonal elements of $\Sigma$, such that if $|\Sigma_{ii}| \leq \epsilon$, then $\Sigma_{ii}$ is set to zero, otherwise set $\Sigma_{ii} = \Sigma_{ii} - \epsilon \cdot \operatorname{sgn}(\Sigma_{ii})$. Now let $H = \frac{p_1 R_1^k + p_2 R_2^k}{p_1 + p_2}$ and $p = \frac{p_1 + p_2}{2}$. The update for $L^{k+1}$ becomes:

$$L^{k+1} = \mathcal{D}_{\frac{1}{p}}(H) = P\xi_{\frac{1}{p}}(\Omega)W^T$$

where $H = P\Omega W^T$ is the SVD of $H$.

**Updating $C$:**

Using the same procedure as done before new have,

$$C^{k+1} = \underset{C}{\operatorname{argmin}} \lambda||C||_{1,2} + \langle Z_1^k, M - L - C \rangle + \frac{p_1}{2}||M - L - C||_F^2$$
$$= \underset{C}{\operatorname{argmin}} \frac{\lambda}{p_1}||C||_{1,2} + ||C - (M - L + \frac{Z_1^k}{p_1})||_F^2$$
$$= \operatorname{prox}_{\frac{\lambda}{p_1}||C||_{1,2}}(M - L^{k+1} + \frac{Z_1^k}{p_1})$$

the proxmity operator of the $||C||_{1,2}$ function is the column-wise soft-thresholding operator. Which is defined by $\zeta_\epsilon(C)$, such that if $||C_i||_2 \leq \epsilon$ ($C_i$ is the ith column of $C$) set $C_i = 0$, otherwise set $C_i = C_i - \epsilon \cdot C_i/||C_i||_2$. Now the update for $C^{k+1}$ becomes:

$$C^{k+1} = \zeta_{\frac{\lambda}{p_1}}(M - L^{k+1} + \frac{Z_1^k}{p_1})$$

**Updating $Q$:**

$$Q^{k+1} = \underset{Q}{\operatorname{argmin}} \gamma \operatorname{tr}(Q\Phi Q^T) + \langle Z_2, Q - L \rangle + \frac{p_2}{2}||Q - L||_F^2$$
$$= \underset{Q}{\operatorname{argmin}} \gamma \operatorname{tr}(Q\Phi Q^T) + \frac{p_2}{2}||Q - (L^{k+1} - \frac{Z_2^k}{p_2})||_F^2.$$

This is a differentiable and convex function, thus finding the first derivative and equation it to zero finds a closed form solution for $Q^{k+1}$.

$$Q^{k+1} = p_2(L^{k+1} - \frac{Z_1^k}{p_2})(\alpha\Phi + p_2 I)^{-1}. \tag{3}$$

## 0.2 Comparing Outlier Detection Performance of GOP and RPCAG

We will show the difference in outlier detection performance of Graph regualrized Outlier Pursuit (GOP) and the Robust PCA on Graphs (RPCAG) formulated by [3]. We will use the 30 randomly sampled instances of the breast cancer data and the single cell dataset. We will compare outlier detection using the $\hat{C}$ method, by sorting the $l_2$ norms of the columns of $\hat{C}$ and record the number of false positives before all known outliers are found. For both datasets we wil first filter the genes to retain the 200 most variable genes across samples. Figure 1 shows that GOP finds less false positives compared to RPCAG on both datasets.

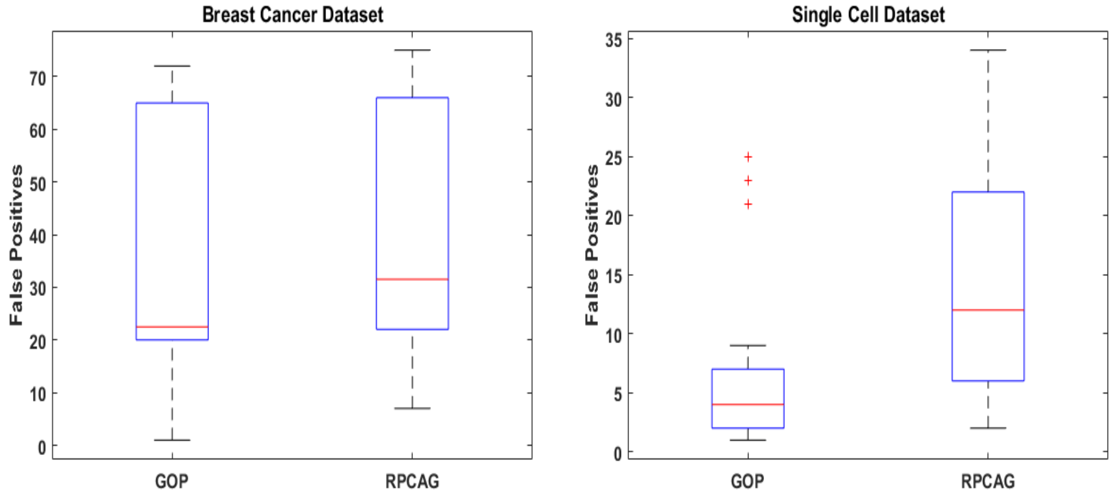


Figure 1: Boxplots comparing the number of detected false positives recorded to detect all outliers in the 30 instances of both the breast cancer and single cell dataset. We can see that the GOP boxplot has less median false positives and a much narrower range when applied to the single cell dataset.

## 0.3 Tuning $\lambda$ for OP and GOP for the Colon Cancer Dataset

We will show how $\lambda$ is tuned for the colon cancer dataset. The procedure is the same for the other datasets used in this study. To tune $\lambda$ for OP we perform a parameter search and search for the optimal value of $\lambda$. For each $\lambda$ value we solve the OP problem and we will use the $\hat{L}$ method to detect outliers as explained in Section 2(e) in the manuscript. A parameter search is performed on $\lambda$ from 0.1 to 0.8 and the rank of $\hat{L}$ is recorded at each step. Figure 2(a) shows that the most stable rank for $\hat{L}$ is one and three. Therefore we refine the range of $\lambda$ from 0.2 to 0.5 and, we record the number of outliers recorded using the $\hat{L}$ method, as shown in Figure 2(b). We need to state that outliers need to be a small fraction of the total dataset it is best chosen to be less than 25 % of the data. Therefore we choose a suitable $\lambda$ to be 0.46 as it gives the smallest number of outliers in the refined range. This gives 9 outlier points, after inspecting their labels, 4 are true outliers and 5 are false positives. To tune $\lambda$ for GOP we use the same procedure as done for OP, we will solve the GOP problem for a range of $\lambda$, from 0.1 to 3, and find the number of outliers for each $\lambda$. Figure 2(c) shows that 4 and 2 outliers are detected over this range of $\lambda$. The most stable rank of $\hat{L}$ is 1 and it detects 4 samples, which means that they can be confidently chosen as being outliers. To be able to visualize in a two-dimensional space we choose a suitable $\lambda$ value as being equal to 1.168. Which gives 4 outliers and a rank of $\hat{L}$ being equal to 2. After finding optimal $\lambda$ we noticed that the number of outliers and the rank of $\hat{L}$ is robust to the value of $\alpha$. Therefore we can choose for simplicity $\alpha$ being equal to 1.

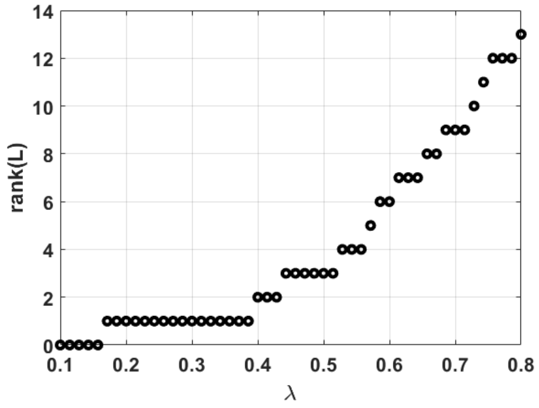


(a)

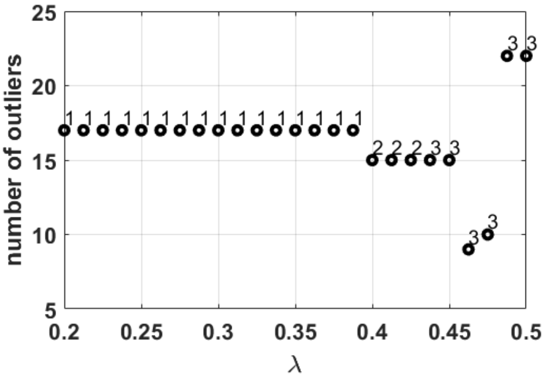


(b)

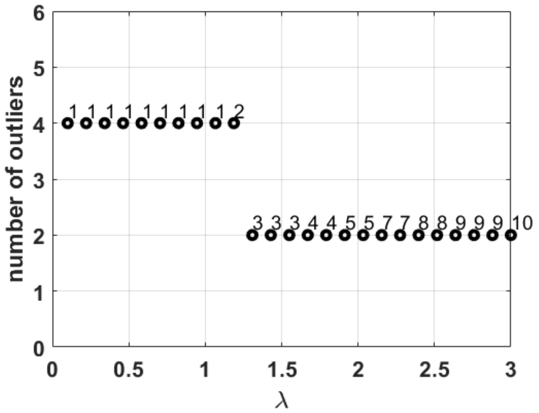


(c)

Figure 2: (a) Rank of recovered low rank matrix by OP versus regularization parameter $\lambda$. Figure shows that the most stable rank for $\hat{L}$ is 1 and 3. Therefore, we can refine the $\lambda$ search space. (b) Refined $\lambda$ search space from 0.2 to 0.5. The labels on the circles are the rank of $\hat{L}$ for a specific $\lambda$. We choose optimal $\lambda$ to be 0.46 which gives the smallest number of outliers, in this case 9 outliers. (c) $\lambda$ vs number of outliers detected. The rank of recovered $\hat{L}$ for each $\lambda$ is shown as the number above each circle. We choose $\lambda$ that gives 4 outliers and a rank of 2.

## 0.4 Boxplot Outlier Detection Method on Different Dimensions

The Boxplot method used for detecting outlier samples in the manuscript has been applied on the TCGA breast cancer dataset filtered to its 200 most variable genes and the cell cycle single cell dataset filtered to its 70 most variable genes. In the Boxplot outlier detection algorithm a sample is labelled as an outlier if the Boxplot method flags a number of outlier genes greater than or equal to a pre-defined threshold. The percentage of false positives encountered to detect all known outliers is 100 % for both datasets. The reason for this is that some of the known outlier samples have no

outlier genes flagged by the Boxplot method. This makes the threshold for flagging all the known outlier samples equal to zero. Therefore, giving 100 % of false positives for all the 30 instances of both dataset. This is seen in Figure 3 and 6 of the main manuscript. To get a better performance from the Boxplot method we increase the number of genes used. This will give a higher possibility to get a greater number of outlier genes per sample. The results for both the TCGA and single cell dataset are shown in figure 3. From Figure 3 (a) we can see that in the breast cancer dataset the Boxplot method had a greater range of false positives when increasing the number of genes. From Figure 3 (b) we can see that the Boxplot method fails to give better performance even when increasing the number of genes present in the data.

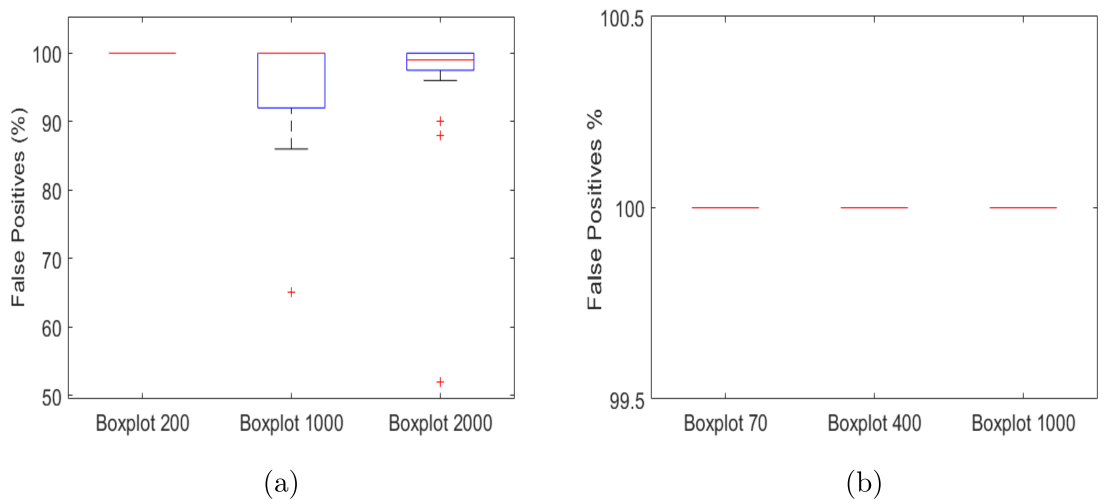


Figure 3: (a) Boxplot outlier detection method applied to breast cancer dataset after filtering 200, 1000, 2000 most variable genes. (b) Boxplot outlier detection method applied to single cell dataset after filtering 70, 400, 1000 most variable genes.

## 0.5 GOP and OP convergence

For both GOP and OP algorithms we inspect the convergence of their objective function. Figure 4 and Figure 5 shows the convergence of GOP and OP on the colon cancer, breast cancer, and single cell datasets.

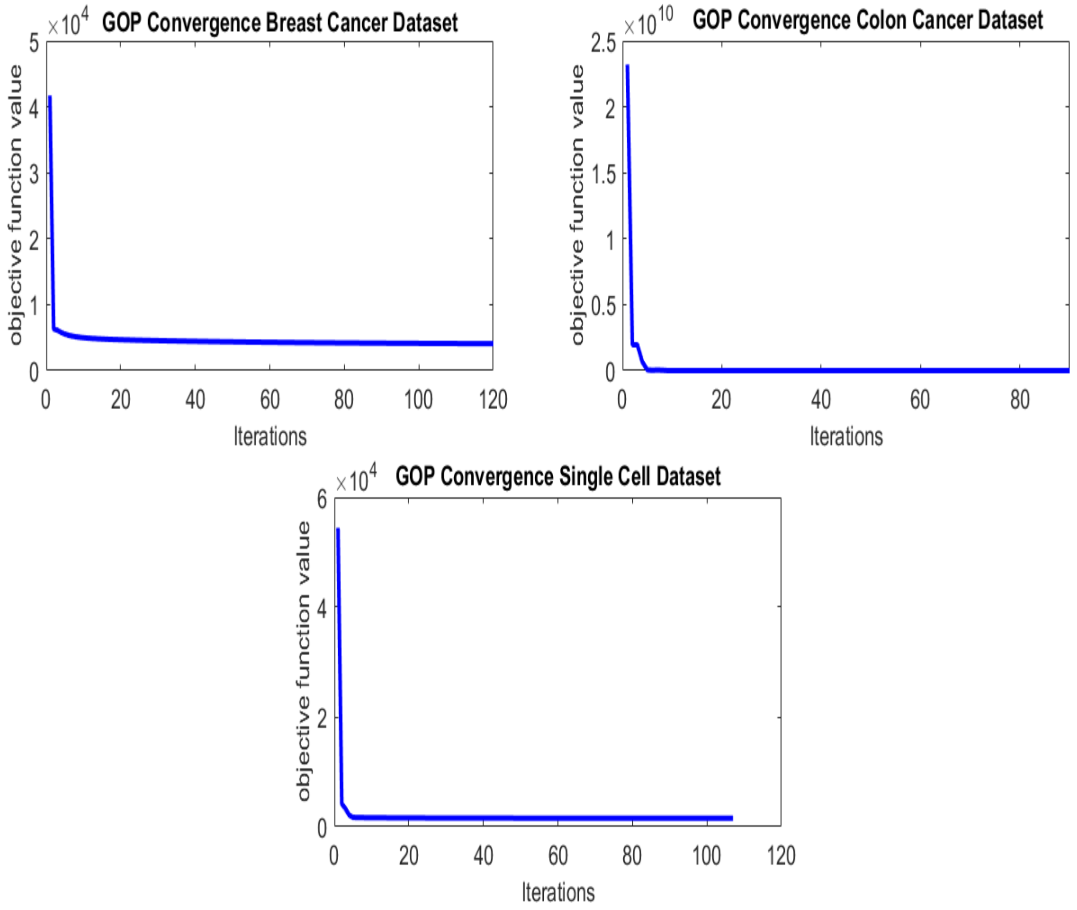


Figure 4: GOP objective function value with repect to number of iterations. The figure shows that the ADMM algorithm formulation for GOP is able to minimizing the objective function.

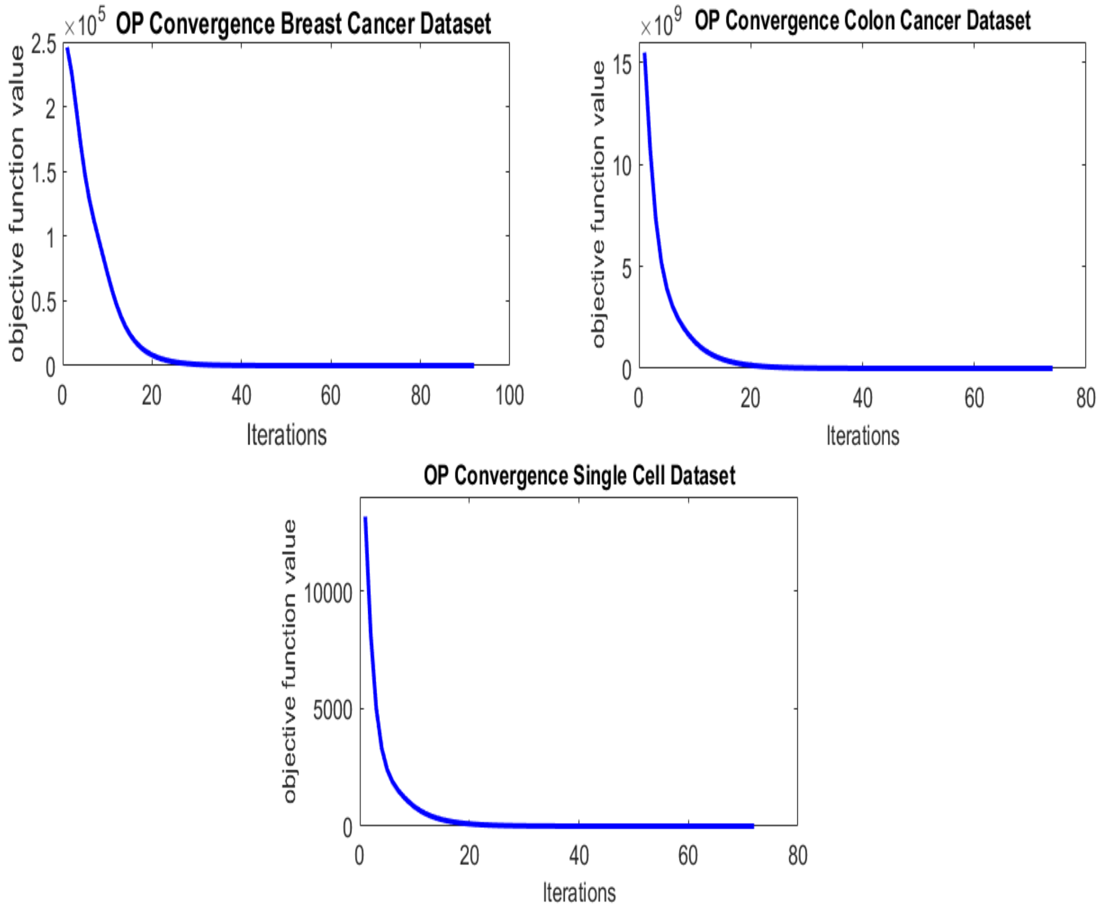


Figure 5: OP objective function value versus number of iterations. We can see from the figure that OP objective function is minimized by the APG algorithm.

# Bibliography

[1] Jian-Feng Cai, Emmanuel J Candès, and Zuowei Shen. A singular value thresholding algorithm for matrix completion. *SIAM Journal on Optimization*, 20(4):1956–1982, 2010.

[2] Patrick L Combettes and Jean-Christophe Pesquet. Proximal splitting methods in signal processing. In *Fixed-point algorithms for inverse problems in science and engineering*, pages 185–212. Springer, 2011.

[3] Nauman Shahid, Vassilis Kalofolias, Xavier Bresson, Michael Bronstein, and Pierre Vandergheynst. Robust principal component analysis on graphs. In *Proceedings of the IEEE International Conference on Computer Vision*, pages 2812–2820, 2015.
